# Tetraarsenictetrasulfide and Arsenic Trioxide Exert Synergistic Effects on Induction of Apoptosis and Differentiation in Acute Promyelocytic Leukemia Cells

**DOI:** 10.1371/journal.pone.0130343

**Published:** 2015-06-25

**Authors:** Shuping Wang, Min Zhou, Jian Ouyang, Zhirong Geng, Zhilin Wang

**Affiliations:** 1 State key Laboratory of Coordination Chemistry, School of Chemistry and Chemical Engineering, Collaborative Innovation Center of Advanced Microstructures, Nanjing University, Nanjing, 210093, China; 2 Department of Hematology, DrumTower Hospital of Medical School, Nanjing University, Nanjing, 210093, China; National Cheng Kung University, TAIWAN

## Abstract

Since arsenic trioxide (As^3+^) has been successfully used in the treatment of acute promyelocytic leukemia (APL), its adverse effects on patients have been problematic and required a solution. Considering the good therapeutic potency and low toxicity of tetraarsenictetrasulfide (As_4_S_4_) in the treatment of APL, we investigated the effects of combining As_4_S_4_ and As^3+^ on the apoptosis and differentiation of NB4 and primary APL cells. As_4_S_4_, acting similarly to As^3+^, arrested the G_1_/S transition, induced the accumulation of cellular reactive oxygen species, and promoted apoptosis. Additionally, low concentrations of As_4_S_4_ (0.1–0.4 μM) induced differentiation of NB4 and primary APL cells. Compared with the As_4_S_4_- or As^3+^-treated groups, the combination of As_4_S_4_ and As^3+^ obviously promoted apoptosis and differentiation of NB4 and primary APL cells. Mechanistic studies suggested that As_4_S_4_ acted synergistically with As^3+^ to down-regulate Bcl-2 and nuclear factor-κB expression, up-regulate Bax and p53 expression, and induce activation of caspase-12 and caspase-3. Moreover, the combination of low concentrations of As_4_S_4_ and As^3+^ enhanced degradation of the promyelocytic leukemia-retinoic acid receptor α oncoprotein. In summary, As_4_S_4_ and As^3+^ synergistically induce the apoptosis and differentiation of NB4 and primary APL cells.

## Introduction

Acute promyelocytic leukemia (APL) is an M3 subtype of acute myeloid leukemia [1]. The typical characteristic of APL is the specific chromosomal translocation t(15;17) (q22;q21), which induces the expression of the promyelocytic leukemia-retinoic acid receptor α (PML-RARα) oncoprotein [1–3]. Two drugs, all-*trans* retinoic acid and arsenic trioxide (As^3+^), have hitherto been successfully used in the treatment of APL [4–6]. At high concentrations (0.5–2.0 μM), As^3+^ triggers apoptosis, and at low concentrations (0.1–0.5μM) it induces partial differentiation of APL cells [7]. Mechanistic studies have suggested that As^3+^ promotes apoptosis and differentiation of APL cells by inducing degradation of the PML-RARα oncoprotein [8]. However, As^3+^ organ injury, especially to the liver and kidneys, causes significant pain to patients [9,10]. Methylation of As^3+^ can induce the accumulation of reactive oxygen species (ROS) and generate more toxic monomethylarsonous and dimethylarsinous acids [11–14]. Currently, combination therapy is widely used in cancer treatment. Therefore, combination therapy for APL treatment can enhance As^3+^ therapeutic potency and reduce its adverse effects.

In addition to As^3+^, realgar is another inorganic form of arsenic that has been used in traditional Chinese medicine for many years [15,16]. Compared with As^3+^, realgar has a positive therapeutic reputation and reduced toxicity [17]. Lu *et al*. have reported that when used alone, the major constituent of realgar, tetraarsenic tetrasulfide (As_4_S_4_), showed high efficiency and safety in all stages of APL [18]. Wang *et al*. showed that the combination of As_4_S_4_, indigo and naturalis can promote the differentiation of APL cells, induce degradation of the PML-RARα fusion protein, and arrest the cell cycle at G_1_/G_0_ [19]. However, the molecular mechanism of As_4_S_4_ potency in APL treatment is unclear. Moreover, realgar is a mixture that contains up to approximately 10% trivalent arsenicals [18], and both bivalent and trivalent arsenicals may contribute to the therapeutic potency of realgar. Clarifying the mechanism of action of As_4_S_4_ and As^3+^ combination on the apoptosis and differentiation of APL cells is necessary.

Apoptosis is the major pathway for drug-induced cancer cell death [20]. Mitochondria-mediated intrinsic apoptosis, death receptor-mediated extrinsic apoptosis and endoplasmic reticulum stress-mediated apoptosis are the three predominant apoptosis pathways and are regulated by a series of apoptotic factors [21]. Of these factors, Bcl-2 family members [22], nuclear factor-κB (NFκB) [23], p53 tumor suppressor [24], caspase-12 and caspase-3 play key roles in As^3+^-induced apoptosis [25]. Although the function of PML is unclear, degradation of the PML-RARα oncoprotein contributes to apoptosis and differentiation of APL cells [8,26]. These apoptotic factors, as well as the PML-RARα fusion protein, may be important for clarifying the mechanism of As_4_S_4_-induced apoptosis and differentiation in APL cells.

The NB4 cell line is a unique APL-derived cell line that expresses the PML-RARα oncoprotein [27]. In this work, we found that As_4_S_4_ and As^3+^ exerted synergistic effects on the apoptosis and differentiation of NB4 and primary APL cells. Multiple pathways were involved in As_4_S_4_ and As^3+^-induced apoptosis. As_4_S_4_ and As^3+^ acted synergistically to promote apoptosis of NB4 cells by up-regulating p53 expression, enhancing the mitochondria-mediated intrinsic pathway, enhancing the endoplasmic reticulum stress-mediated pathway, and inhibiting the NFκB signaling pathway. Moreover, low doses of As_4_S_4_ could be combined with As^3+^ to enhance degradation of the PML-RARα oncoprotein and promote NB4 and primary APL cell differentiation through the retinoic acid-signaling pathway.

## Materials and Methods

Caution: Due to the potential risk of arsenic compounds, safeguards should be implemented [11–14].

### Reagents

High purity As_4_S_4_ was obtained from Yiji industry (Shanghai, China). NaAsO_2_, bovine serum albumin (BSA), anti-PML rabbit mAb and anti-caspase-12 rabbit mAb were purchased from Sigma-Aldrich (St. Louis, MO, USA). Anti-Bcl-2 (50E3) rabbit mAb, anti-Bax (D2E11) rabbit mAb, anti-NFκB p65 (D14E12) XP rabbit mAb, anti-caspase-3 (8G10) rabbit mAb and anti-p53 (7F5) rabbit mAb were obtained from Cell Signaling Technology (Boston, MA, USA). Anti-β-actin mouse mAb was purchased from Beyotime (Nantong, China). FITC anti-human CD11b antibody was obtained from BioLegend (San Diego, CA. USA).

### Cell culture and growth assay

Human NB4 leukemia cells were purchased from SXBIO Biotech (Shanghai, China). Human primary APL cells were separated from the bone marrow of four primary APL patients acquired from DrumTower Hospital (Nanjing, China) by Ficoll-Hypaque density centrifugation as reported in the previously published article [28]. Written informed consent was obtained from individual subjects. For this case we did not seek approval of the Ethics Committee of Drum Tower Hospital and did not obtain a waiver from the Ethics Committee because the bone marrow was part of that acquired for clinical diagnosis and destroyed after this experiment. NB4 cells were cultured in RPMI-1640 (KeyGEN Biotech, China) with 10% fetal bovine serum (FBS) at 37°C under a 5% CO_2_ atmosphere. The fresh primary APL cells were cultured in RPMI-1640 with 15% FBS. Trypan blue exclusion was used to determine viability of NB4 and primary APL cells after 48 h and 96 h of culture [28]. The effects of As_4_S_4_ and As^3+^ on cell growth were determined using the WST-1 cell proliferation assay kit (KeyGEN Biotech, China). In brief, 4×10^4^ cells/ml were seeded into a 96-well culture plate and treated with various concentrations of As_4_S_4_, As^3+^, or their combination for 48 h. Untreated cells served as controls [28,29].

### Calculation of combination index (CI)

The CI values were calculated by equation (1): CI = [D]_1_/[D_x_]_1_ + [D]_2_/[D_x_]_2_ + α[D]_1_[D]_2_/[D_x_]_2_[D_x_]_2_ [30]. Here, [D_x_]_1_ and [D_x_]_2_ respectively represent the concentrations of As^3+^ and As_4_S_4_ alone, resulting in growth inhibition of NB4 and primary APL cells (in x%). [D]_1_ and [D]_2_ are the concentrations of As^3+^ and As_4_S_4_ when they are used in combination to inhibit the cell growth at same percentage (x%). When the two drugs are assumed to be non-exclusive, the value of α is 1; when the two drugs are assumed to be exclusive, the value of α is 0. CI = 1 indicate an additive effect; CI < 1indicate a synergistic effect; CI > 1 indicate an antagonism effect [30].

### Analysis of apoptosis

The proportions of apoptotic cells were measured with the Annexin V-FITC and propidium iodide (PI) apoptosis detection kit (KeyGEN Biotech, China) using flow cytometry [28,29,31]. NB4 and primary APL cells were treated with 2.0 μM As_4_S_4_, 2.0 μM As^3+^, or 1.0 μM As_4_S_4_ and 1.0 μM As^3+^ for 48 h. After treatment, the cells were washed twice with Ca^2+^-free phosphate buffer (PBS), stained with Annexin V-FITC and PI in the dark at room temperature for 15 min, and detected using a BD LSRL Fortessa flow cytometer. The percentages of apoptotic cells were analyzed using the BD FACSDiva software.

### Analysis of cell cycle distribution

The effects of As_4_S_4_ and As^3+^ on cell cycle distribution were determined using a PI cell cycle detection kit (KeyGEN Biotech, China) [28]. After treatment with As_4_S_4_ (2.0 μM), As^3+^ (2.0 μM), or a combination of 1.0 μM As_4_S_4_ and 1.0 μM As^3+^ for 48 h, NB4 and primary APL cells were collected, washed with Ca^2+^-free PBS and fixed with 70% ethanol at 4°C for 16 h. The fixed cells were then digested in PBS containing 0.5 mg/ml RNase (Sigma-Aldrich, USA) at 37°C for 30 min and stained with 0.05 mg/ml PI in the dark at room temperature for 30 min. Data on the cell cycle distribution were determined using the ModFit LT 3.3 software.

### Analysis of cellular ROS levels using 2’,7’-dichlorodihydrofluorescein diacetate (DCFH-DA)

Cellular ROS levels were assayed using DCFH-DA. DCFH-DA can be hydrolyzed to DCFH by esterases and oxidized to 2’,7’-dichlorofluorescein (DCF) by cellular ROS [32]. Thus, the DCF fluorescence intensity is positively correlated with cellular ROS levels. After treatment with As_4_S_4_ (0.5, 1.0 and 2.0 μM), As^3+^ (2.0 μM), or a combination of 1.0 μM As_4_S_4_ and 1.0 μM As^3+^ for 24 h and 36 h, NB4 and primary APL cells were washed twice with PBS and then incubated in RPMI-1640 medium containing 10.0 μM DCFH-DA at 37°C for 30 min. Excess probe was washed out with PBS, and the percentages of DCF-positive cells were detected by flow cytometry and analyzed using FlowJo.7.6.

### Analysis of mRNA by RT-PCR

RNAiso Plus (Takara-Bio, Japan) was used to extract total RNA from NB4 and primary APL cells. The concentration and purity of the isolated total RNA were determined by trace nucleic acid protein measurement instrument (NanoDrop ND-1000) [28,29]. 2.0 μg of total RNA was reverse-transcribed to cDNA, and 2.0 μL of transcribed cDNA was used for PCR amplification with specific primers. After initial denaturing at 94°C for 5 min, thirty cycles of 30 s denaturation at 94°C; 30 s annealing at 52°C (β-actin), 57°C (heme oxygenase-1 (HMOX1), Bax, Bcl-2, and NFκB-3), or 51°C (caspase-3); and 30 s extension at 72°C were performed. The PCR products were separated on 1% agarose gels containing ethidium bromide. The separated bands were imaged on a Gel Doc XR System (Bio-Rad). The primer sequences are shown in Table 1.

**Table 1:** Primer sequences for the apoptosis factors in RT-PCR.

Name	Primer sequence	
HMOX1	sense	5′-CTTTGAGGAGTTGCAGGAGC-3′
	antisense	5′-TGTAAGGACCCATCGGAGAA-3′
Bax	sense	5′-TGACGGCAACTTCAACTGGG-3′
	antisense	5′-AGCACTCCCGCCACAAAGA-3′
Bcl-2	sense	5′-GGGAGGATTGTGGCCTTCTT-3′
	antisense	5′-GGCCAAACTGAGCAGAGTCTTC-3′
NFκB-3	sense	5′-ACTACGAGGGACCAGCCAAGA-3′
	antisense	5′-CGCAGCCGCACTATACTCA-3′
Caspase-3	sense	5′-GTGGAATTGATGCGTGATG-3′
	antisense	5′-AACCAGGTGCTGTGGAGTA-3′
β-Actin	sense	5′-GACCTGACTGACTACCTC-3′
	antisense	5′-TCTTCATTGTGCTGGGTGC-3′

### Protein analysis by western-blot

After treatment with As_4_S_4_, As^3+^ or a combination, NB4 and primary APL cells were collected, washed twice with PBS, and then lysed in ice-cold RIPA cell lysis buffer (Beyotime, China) containing 1.0 mM PMSF for 60 min to extract total cellular protein [28,29]. The concentration of total protein was determined using the BCA protein quantification kit (Beyotime, China). 25.0 μg of total protein from each sample was separated by sodium dodecyl sulfate-polyacrylamide gel electrophoresis and transferred onto a PVDF membrane (Millipore, USA).The membrane was then blocked with 5% skim milk at room temperature for 60 min and sequentially incubated with primary and secondary antibodies. Proteins on the PVDF membrane were visualized using chemiluminescent HRP substrate (Millipore, USA). The band intensities were corrected using the β-actin intensities. All experiments were repeated at least three times.

### Analysis of cell differentiation

We analyzed NB4 and primary APL cell differentiation with an FITC anti-human CD11b antibody using flow cytometry [19]. After treatment with As_4_S_4_, As^3+^, or a combination for 96 h, cells were harvested, washed twice with PBS and counted. A total of 1.0×10^6^ cells in 100 μl PBS were incubated with 20 μl FITC anti-human CD11b antibody at 4°C for 30 min. Excess antibody was washed out, and the percentages of FITC-CD11b-positive cells were analyzed using FlowJo.7.6.

### Statistical analysis

Two-tailed Student’s *t*-tests were performed for comparisons of two groups, and P<0.05 was considered to be statistically significant.

## Results

### As_4_S_4_ enhances As^3+^-inhibition of NB4 and primary APL cell growth

**Fig 1:**
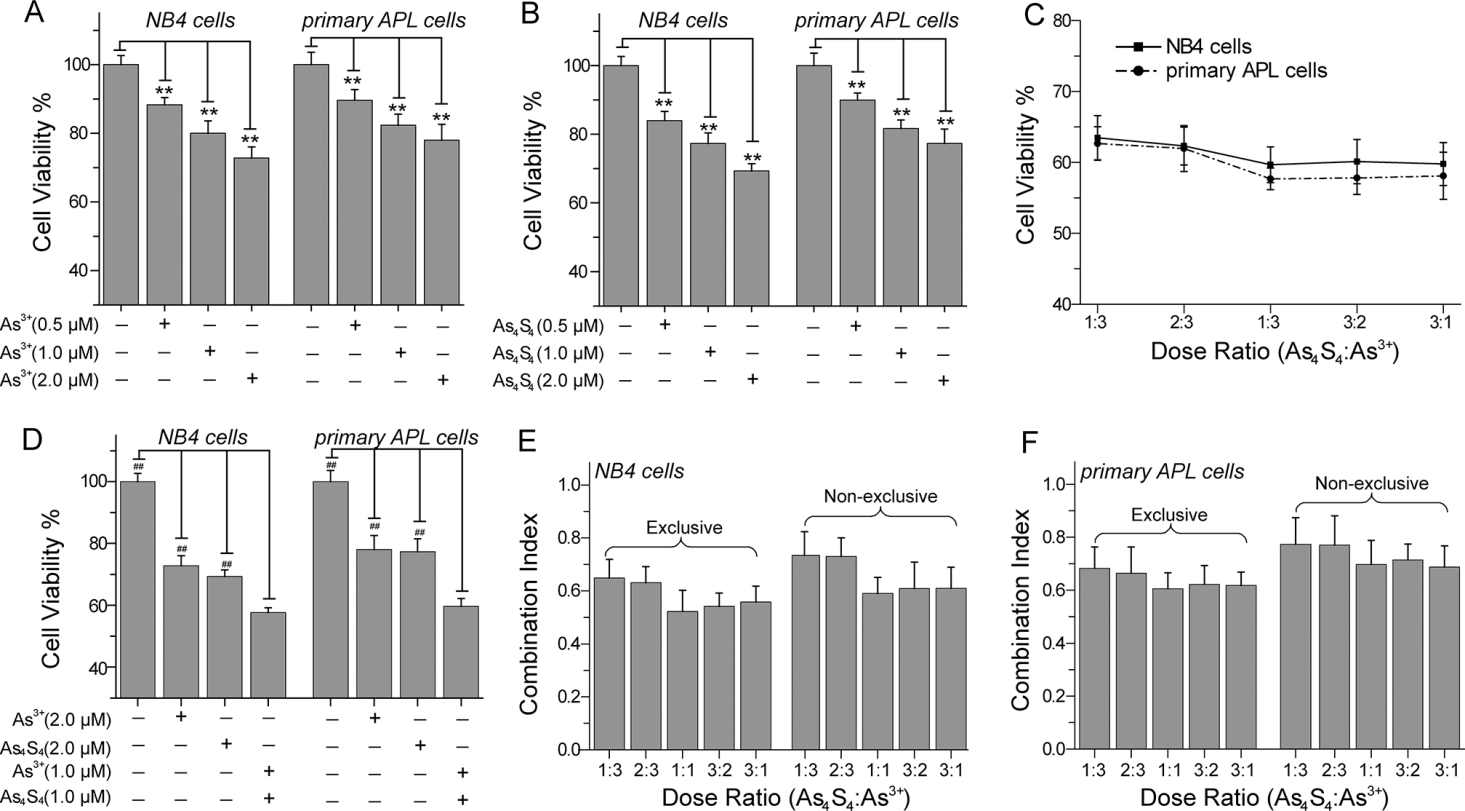
The effects of combining As^3+^ and As_4_S_4_ on the growth of NB4 and primary APL cells. (A) The effects of As^**3+**^ on cell viability. (B) The effects of As_**4**_S_**4**_ on cell viability. (C&D) The combined effects of As^**3+**^ and As_**4**_S_**4**_ on cell viability. (E) CI of concurrent treatment with As^**3+**^ and As_**4**_S_**4**_ in NB4 cells. (F) CI in primary APL cells. CI<1.0 indicated a synergistic effect. The viability of NB4 and primary APL cells were determined by WST-1 cell proliferation assay kit after 48 h of treatment. *Error bars* represent the S.D. from the mean of three separate experiments. **P<0.01 compared with the control. ^##^P<0.01 compared with As^3+^ and As_4_S_4_ combination treated cells.

The viability of NB4 and primary APL cells was determined by trypan blue exclusion [28]. Following 48 h of treatment, 97.5% of NB4 cells and 97.0% of primary APL cells were viable. The effects of As_4_S_4_, As^3+^, or their combination on NB4 and primary APL cell proliferation were analyzed using the WST-1 cell proliferation assay. After 48 h of treatment, As^3+^ and As_4_S_4_ obviously inhibited the growth of NB4 and primary APL cells (Fig 1A and 1B). The inhibitory potency of As^3+^ and As_4_S_4_ on cell growth increased with the increasing of concentration from 0.5 μM to 2.0 μM (Fig 1A and 1B). Subsequently, we investigated the combination effects of As^3+^ (0.5–1.5 μM) and As_4_S_4_ (0.5–1.5 μM) at fixed dose ratio (As_4_S_4_/As^3+^ were 1/3, 2/3, 1/1, 3/2, and 3/1) on the growth of NB4 and primary APL cells (Fig 1C). In our tested range of dose ratios, combining As_4_S_4_ and As^3+^ obviously inhibited the growth of NB4 and primary APL cells (Fig 1C). Compared with 2.0 μM As^3+^ alone or 2.0 μM As_4_S_4_ alone treated group, the combination of 1.0 μM As^3+^ and 1.0 μM As_4_S_4_ markedly induced the death of NB4 and primary APL cells (Fig 1D). In order to clarify whether As^3+^ and As_4_S_4_ exerted synergistic effects on cell growth, we analyzed CI values of the two drugs at different dose ratios (Fig 1E and 1F). When the dose ratio of As_4_S_4_/As^3+^ was in the range of 1:3 to 3:1, the combination of As^3+^ and As_4_S_4_ yielded a moderate synergistic effect (0.6≤CI<0.8) on the growth of NB4 and primary APL cells (Fig 1E and 1F) [30].

### As_4_S_4_ acts synergistically with As^3+^ to promote the apoptosis of NB4 and primary APL cells

**Fig 2:**
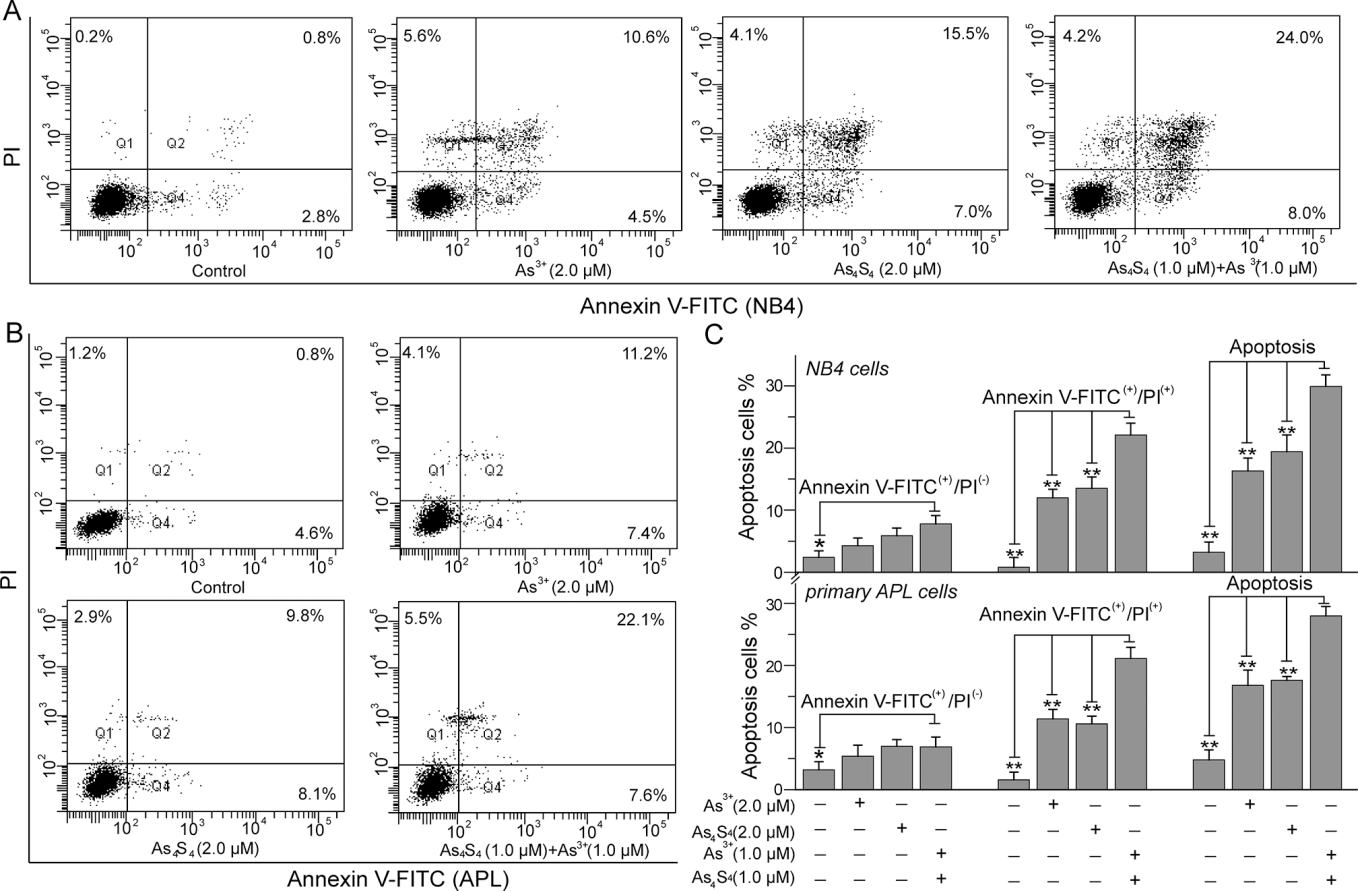
The effects of combining As^3+^ and As_4_S_4_ on the apoptosis of NB4 and primary APL cells. (A) The apoptosis of NB4 cells. (B) The apoptosis of primary APL cells. (C) The percentage of apoptotic cells in NB4 and primary APL cells. After 48 h of treatment, the cells were stained with Annexin V-FITC and PI. Q_**1**_ and Q_**3,**_ represent the dead cells and living cells, respectively. Q_**2**_ and Q_**4**_ were used to calculate the proportion of apoptotic cells. Figures show a representative experiment of three independent experiments. *P<0.05 and **P<0.01 compared with As^3+^ and As_4_S_4_ combination treated cells.

Cell death comprises two major pathways: apoptosis and necrosis [20]. We investigated the effects of As_4_S_4_ on As^3+^-induced apoptosis in NB4 and primary APL cells by flow cytometry. As shown in Fig 2, Q_2_ and Q_4_ represent the percentages of Annexin V-FITC^(+)^/PI^(+)^ and Annexin V-FITC^(+)^/PI^(-)^ cells, respectively. After 48 h of treatment, both As^3+^ and As_4_S_4_ obviously induced NB4 cell apoptosis (Fig 2A). Compared with the control, the percentage of apoptotic cells in the group treated with 2.0 μM As_4_S_4_ increased from 3.7±1.3% to 19.4±2.6%, and the percentage reached 16.3±2.0% in the 2.0 μM As^3+^-treated group. Subsequently, we investigated the effects of combining 1.0 μM As_4_S_4_ and 1.0 μM As^3+^ on NB4 cell apoptosis. Compared with the groups treated with either 2.0 μM As_4_S_4_ or 2.0 μM As^3+^, the combination of 1.0 μM As_4_S_4_ and 1.0 μM As^3+^ markedly increased the proportion of apoptotic cells to 29.9±1.9% (Fig 2A and 2C). Similarly, As_4_S_4_ acted synergistically effects with As^3+^ on primary APL cell apoptosis (Fig 2B). Compared with the control, 2.0 μM As_4_S_4_ increased the percentage of apoptotic cells from 4.8±1.6% to 17.6±0.7%, and 2.0 μM As^3+^ increased the percentage of apoptotic cells to 16.8±2.6%. Furthermore, 1.0 μM As_4_S_4_ and 1.0 μM As^3+^ acted synergistically to increase the percentage of apoptotic cells to 28.0±1.5% (Fig 2B and 2C). The combination of As_4_S_4_ and As^3+^ synergistically promoted NB4 and primary APL cell apoptosis.

### As_4_S_4_ and As^3+^ act synergistically to arrest the cell cycle in G_0_/G_1_ phase

**Fig 3:**
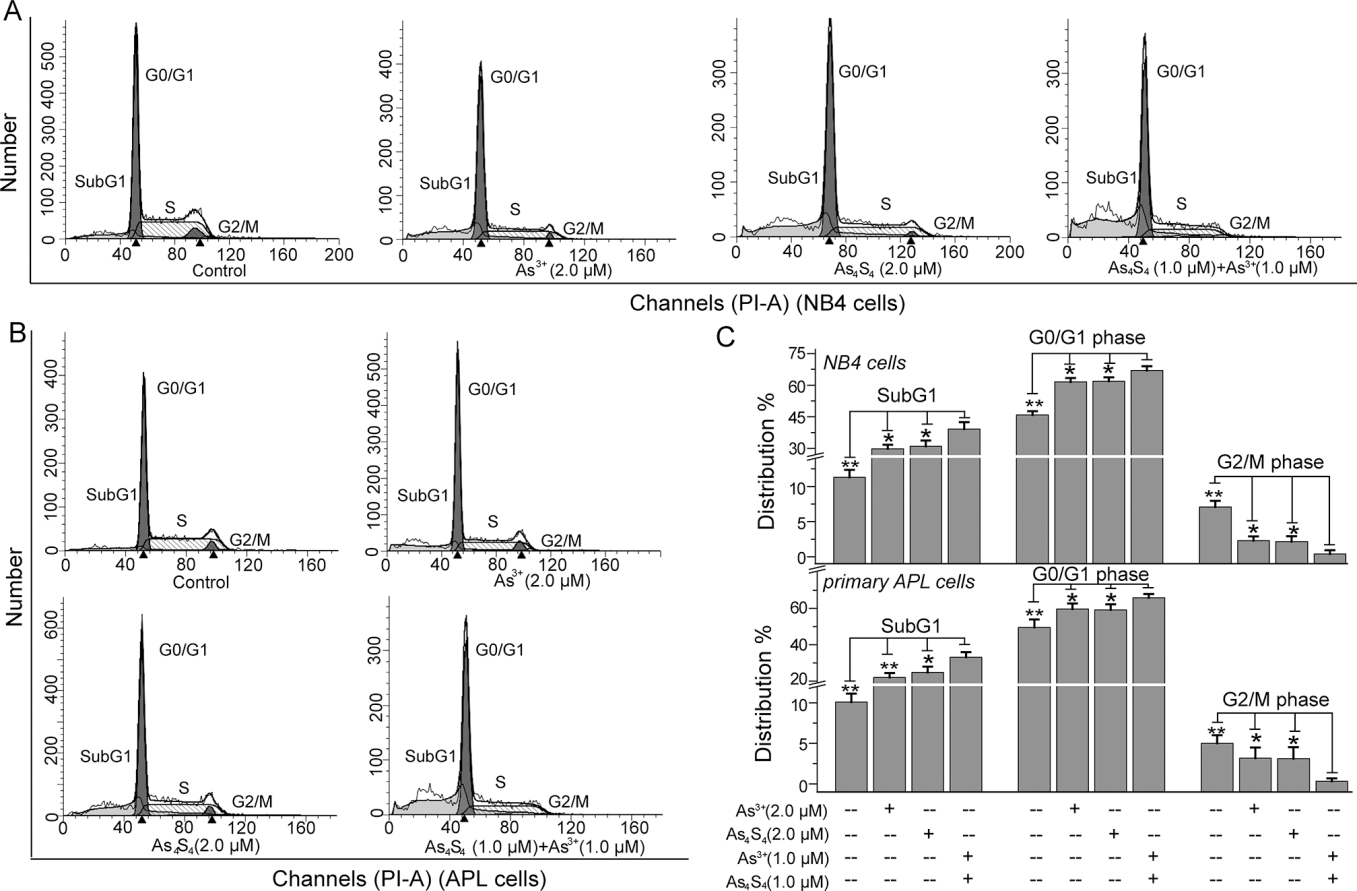
The effects of combining As^3+^ and As_4_S_4_ on cell cycle distribution. (A) Cell cycle distribution in NB4 cells. (B) Cell cycle distribution in primary APL cells. (C) The percentage of cell cycle distribution in each phase. After 48 h of treatment, NB4 and primary APL cells were stained with PI and analyzed by flow cytometry. Figures show a representative experiment of three independent experiments. *P<0.05 and **P<0.01 compared with As^3+^ and As_4_S_4_ combination treated cells.

The cell cycle is a highly regulated process, and aberrations in cell cycle distribution can induce abnormal cell changes such as apoptosis and differentiation [33,34]. The SubG_1_ content represents the percentage of apoptotic cells [35]. In agreement with the results of the Annexin V-FITC and PI staining, As^3+^ and As_4_S_4_ obviously increased the SubG_1_ contents (Fig 3). Furthermore, As_4_S_4_ enhanced As^3+^-induced apoptosis in NB4 and primary APL cells (Fig 3A and 3B). Compared with the groups treated with either 2.0 μM As^3+^ or 2.0 μM As_4_S_4_, combined treatment with 1.0 μM As^3+^ and 1.0 μM As_4_S_4_ for 48 h resulted in the increasing of SubG_1_ content (Fig 3).

As^3+^ inhibited the G_1_/S and S/G_2_ transitions of NB4 and primary APL cells (Fig 3). In NB4 cells, As^3+^ increased the percentage of DNA at G_0_/G_1_ phase from 45.9±1.85% to 61.5±1.9%, and the percentage at G_2_/M phase decreased from 7.1±0.9% to 2.3±0.6% (Fig 3A and 3C). In primary APL cells, As^3+^ increased the percentage of DNA at G_0_/G_1_ phase from 45.9±1.85% to 61.9±1.8%, and the percentage at G_2_/M phase decreased from 7.1±0.9% to 2.1±0.8% (Fig 3B and 3C). As_4_S_4_, acting similarly to As^3+^, increased the percentage of DNA at G_0_/G_1_ phase and decreased the percentage at G_2_/M phase (Fig 3). Compared with 2.0 μM As^3+^ alone or 2.0 μM As_4_S_4_ alone treated group, the combination of 1.0 μM As_4_S_4_ and 1.0 μM As^3+^ obviously blocked the G_1_/S transition, as the DNA content at G_0_/G_1_ phase respectively reached 67.0±2.1% and 65.9±2.0% in NB4 and primary APL cells (Fig 3).

### As_4_S_4_ has no obvious effects on As^3+^-induced ROS accumulation

**Fig 4:**
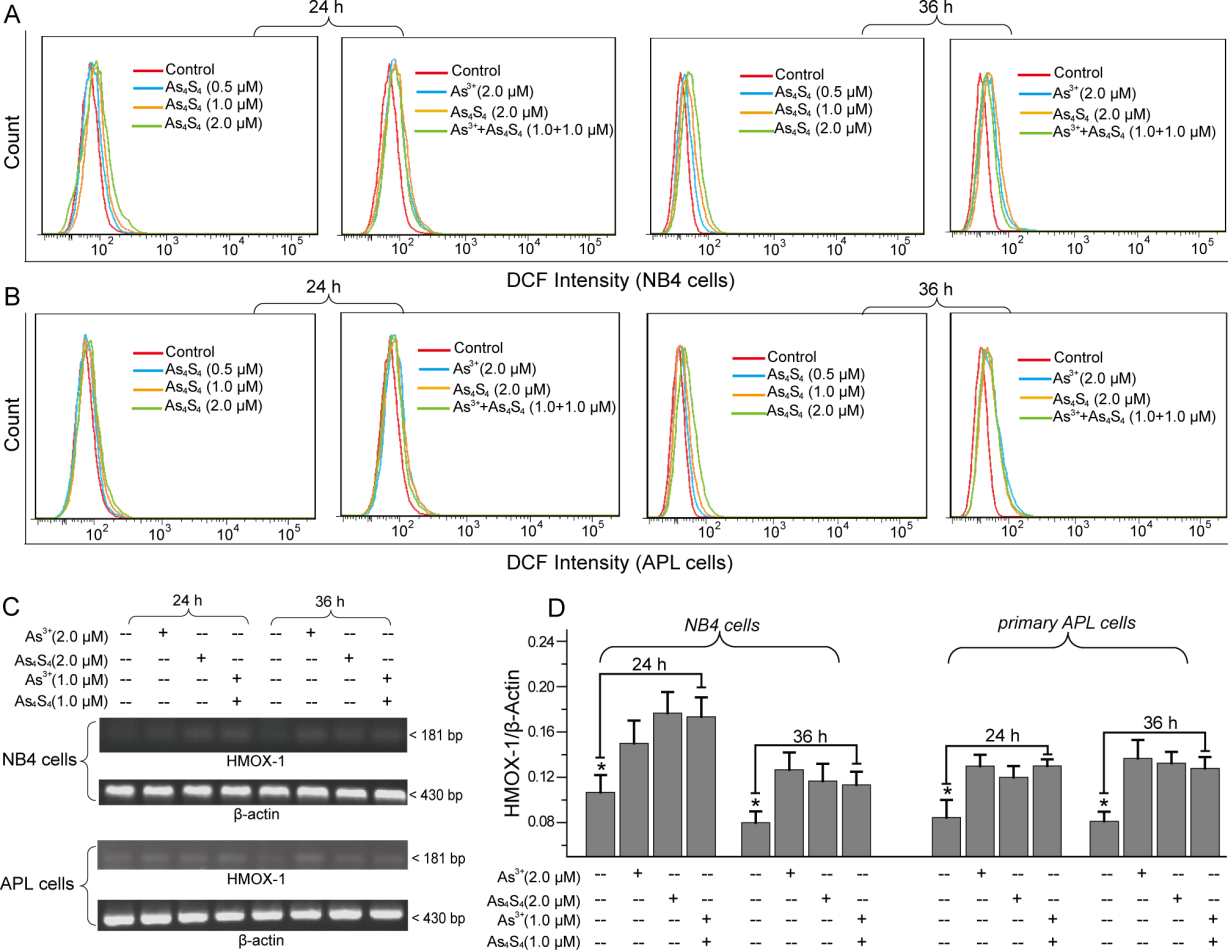
The effects of combining As^3+^ and As_4_S_4_ on cellular ROS accumulation. (A) Cellular ROS were determined with DCFH-DA fluorescence probe in NB4 cells. (B) Cellular ROS in primary APL cells. (C) The effects of As_**4**_S_**4**_ and As^**3+**^ on HMOX1 expression. (D) The percentage of relative HMOX1 intensity obtained by RT-PCR. *Error bars* represent the S.D. from the mean of three independent experiments. *P<0.05 compared with As^3+^ and As_4_S_4_ combination treated cells.

As^3+^-induced apoptosis is related to cellular ROS accumulation [7,24]. To investigate whether As_4_S_4_-induced apoptosis in NB4 and primary APL cells is related to ROS generation, we detected cellular ROS with DCFH-DA fluorescence probe by flow cytometry. The results suggested that As_4_S_4_ obviously increased the percentage of DCF-positive cells, indicating an increase in cellular ROS levels (Fig 4A and 4B) [32]. Compared with the 2.0 μM As^3+^- or 2.0 μM As_4_S_4_-treated groups, the combination of 1.0 μM As^3+^ and 1.0 μM As_4_S_4_ did not obviously increase the percentage of DCF-positive cells (Fig 4A and 4B). We also analyzed the expression of HMOX1, a key oxidative stress response enzyme, to examine the effects of combining As^3+^ and As_4_S_4_ on cellular ROS [36]. RT-PCR analysis showed that 2.0 μM As^3+^ and 2.0 μM As_4_S_4_ up-regulated HMOX1 expression (Fig 4C). However, HMOX1 expression was not significantly different between cells treated with 2.0 μM As^3+^ and cells treated with both 1.0 μM As^3+^ and 1.0 μM As_4_S_4_ (Fig 4C and 4D). Notably, As_4_S_4_ induced ROS accumulation in NB4 and primary APL cells but did not affect As^3+^-induced ROS accumulation.

### As_4_S_4_ and As^3+^ regulate apoptotic factor expression

**Fig 5:**
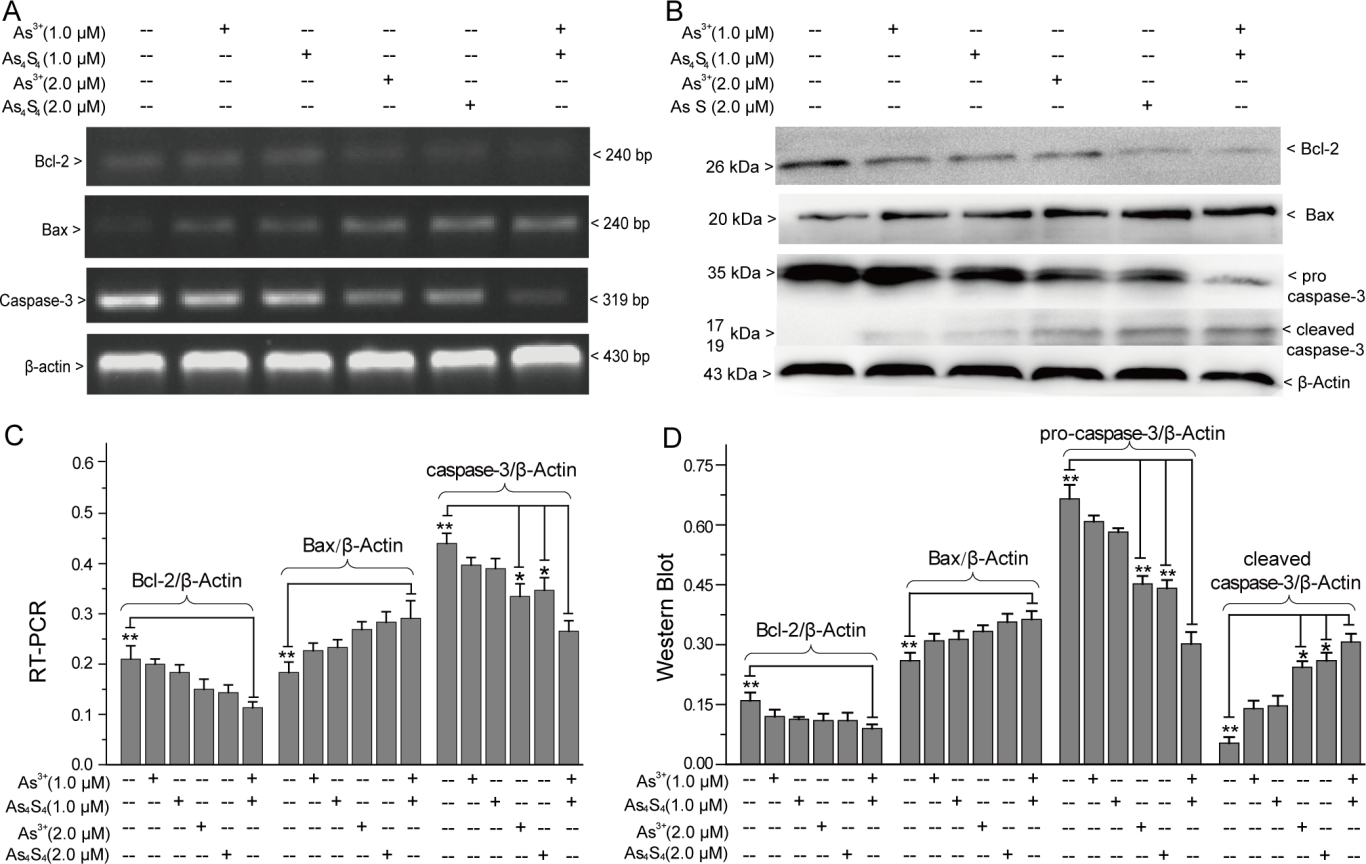
The effects of combining As_4_S_4_ and As^3+^ on mitochondria-mediated apoptosis. (A) RT-PCR analysis of Bax, Bcl-2 and caspase-3 expression. (B) Western-blot analysis of Bax, Bcl-2 and caspase-3 expression. (C) Relative intensity of expression obtained by RT-PCR. (D) Relative intensity expression obtained by western-blot. *Error bars* represent the S.D. from the mean of three separate experiments. *P<0.05 and **P<0.01 compared with As^3+^ and As_4_S_4_ combination treated cells.

Bax, Bcl-2, caspase-3, NFκB, p53 and caspase-12 are the six factors that play key roles in As^3+^-induced cell apoptosis [21–25]. To clarify the mechanism of apoptosis, we investigated the effects of combining As_4_S_4_ and As^3+^ on the expression of these factors. RT-PCR and western-blot analysis suggested that both As_4_S_4_ and As^3+^ up-regulated the expression of pro-apoptotic factor Bax, down-regulated the expression of anti-apoptotic factor Bcl-2, and induced the activation of caspase-3 (Fig 5A and 5B). Compared with 2.0 μM As^3+^ alone or 2.0 μM As_4_S_4_ alone treated group, the combination of 1.0 μM As_4_S_4_ and 1.0 μM As^3+^ enhanced caspase-3 activation. However, their combined effects on Bax and Bcl-2 expression were not obvious (Fig 5C and 5D).

**Fig 6:**
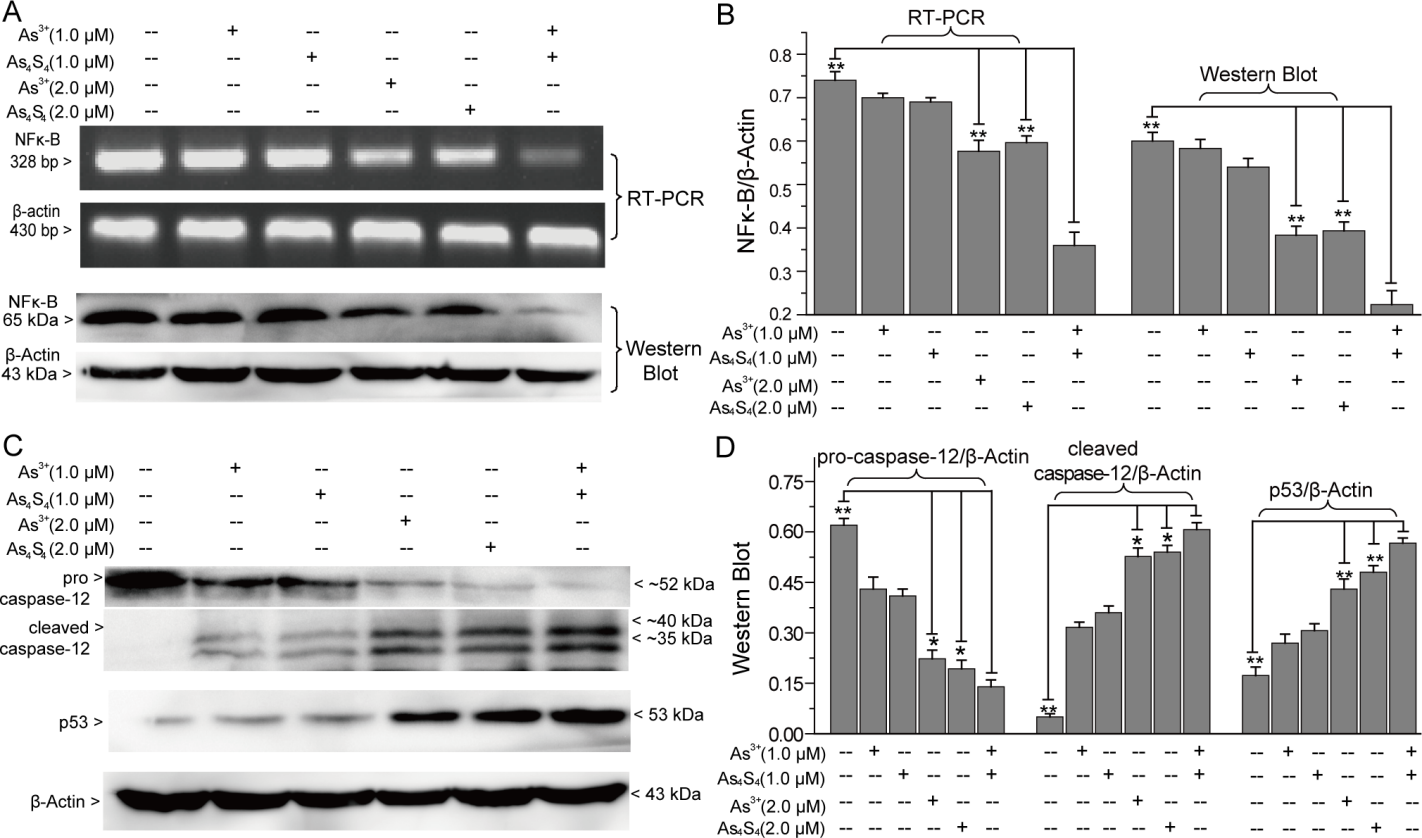
The effects of combining As_4_S_4_ and As^3+^ on NFκB, caspase-12 and p53 expression. (A) RT-PCR and Western-blot analysis of NFκB expression. (B) Relative NFκB intensity obtained by RT-PCR and western-blot. (C) The effects of As_**4**_S_**4**_ and As^**3+**^ on caspase-12 and p53 expression. (D) Relative intensities of caspase-12 and p53 obtained by western-blot. *Error bars* represent the S.D. from the mean of three separate experiments. *P<0.05 and **P<0.01 compared with As^3+^ and As_4_S_4_ combination treated cells.

Subsequently, we investigated the effects of As_4_S_4_ and As^3+^ on NFκB, caspase-12 and p53 expression (Fig 6). As_4_S_4_ inhibited NFκB expression similarly to As^3+^ (Fig 6A and 6B). Moreover, As_4_S_4_ induced caspase-12 activation and promoted p53 tumor suppressor expression (Fig 6C and 6D). Compared with 2.0 μM As^3+^ alone or 2.0 μM As_4_S_4_ alone treated group, the addition of 1.0 μM As_4_S_4_ obviously promoted the regulation of 1.0 μM As^3+^ on NFκB, caspase-12 and p53 (Fig 6).

### Low concentrations of As_4_S_4_ promote As^3+^-induced cell differentiation

**Fig 7:**
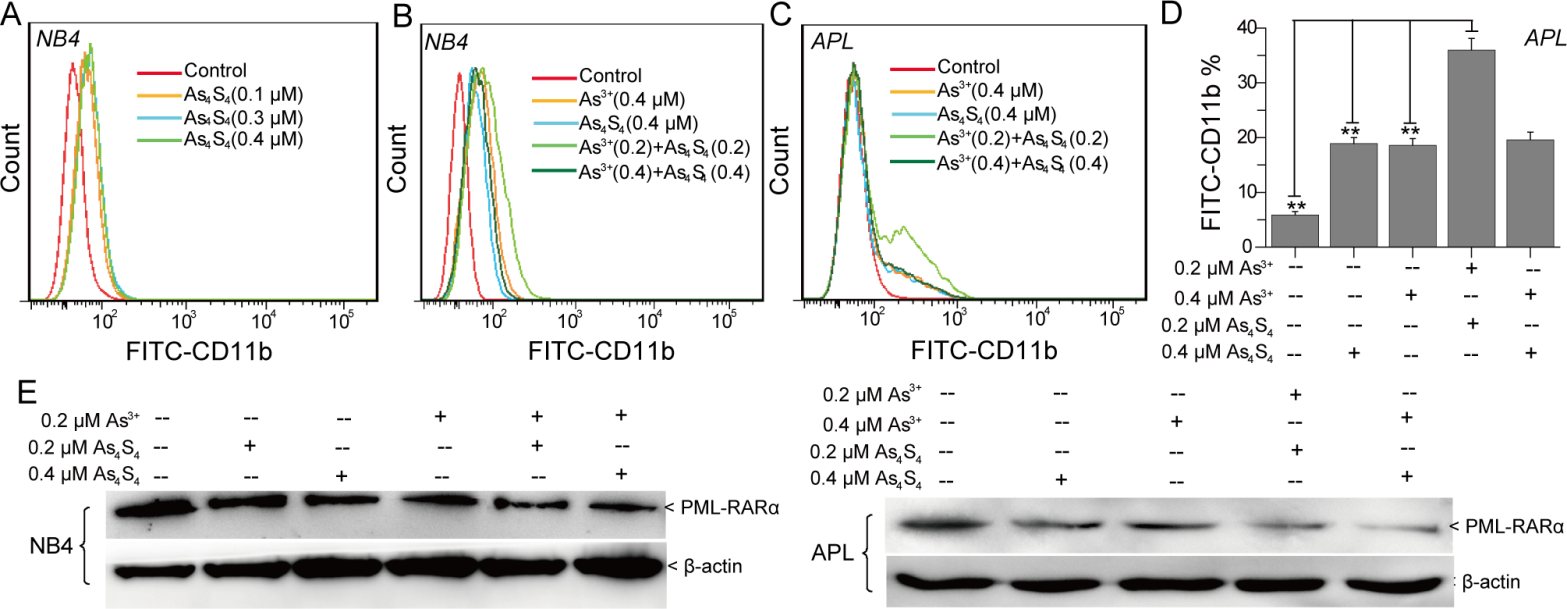
As_4_S_4_ acts synergistically with As^3+^ to affect NB4 and primary APL cell differentiation. (A) The effects of As_**4**_S_**4**_ on CD11b expression in NB4 cells. (B) The effects of As_**4**_S_**4**_ on As^**3+**^-induced differentiation in NB4 cells. (C) The effects of As_**4**_S_**4**_ on As^**3+**^-induced differentiation in primary APL cells. (D) The percentage of FITC-CD11b-positive primary APL cells. (E) Western-blot analysis of PML-RARα expression in NB4 and primary APL cells. The figures show a representative experiment of three independent experiments. **P<0.01 compared with 0.2 μM As^3+^ and 0.2 μM As_4_S_4_ combination treated cells.

Previous research suggested that As_4_S_4_ could induce NB4 cell differentiation [19]. However, the effects of As_4_S_4_ on As^3+^-induced NB4 cell differentiation have not been clarified. Herein, we investigated the effects of combining As_4_S_4_ and As^3+^ on NB4 and primary APL cell differentiation. As shown in Fig 7A, 0.1–0.4 μM As_4_S_4_ induced NB4 cell differentiation. When the concentration of As_4_S_4_ was greater than 0.3 μM, the percentage of CD11b-positive cells did not increase with the increasing of As_4_S_4_ concentration any more (Fig 7A). Compared with 0.4 μM As^3+^ alone- or 0.4 μM As_4_S_4_ alone-treated groups, the combination of 0.2 μM As_4_S_4_ and 0.2 μM As^3+^ obviously promoted NB4 cell differentiation (Fig 7B). Similarly, 0.1–0.4 μM As_4_S_4_ induced primary APL cell differentiation. Compared with the 0.4 μM As^3+^ and 0.4 μM As_4_S_4_ treatments, the percentage of CD11b-positive cells was obviously increased in the group treated with both 0.2 μM As_4_S_4_ and 0.2 μM As^3+^; however, the percentage of CD11b-positive cells was not increased in the group treated with 0.4 μM As_4_S_4_ and 0.4 μM As^3+^ (Fig 7C and 7D). Further investigation suggested that 0.1–0.25 μM As^3+^ and 0.1–0.25 μM As_4_S_4_ synergistically promoted the differentiation of NB4 and primary APL cells (data not show). The formation of the PML-RARα oncoprotein blocks NB4 and primary APL cell differentiation [8]. Therefore, we studied the effects of combining As_4_S_4_ and As^3+^ on PML-RARα expression by western-blot. After 96 h of treatment, 0.2 and 0.4 μM As_4_S_4_ induced PML-RARα oncoprotein degradation. Furthermore, As_4_S_4_ enhanced the As^3+^-induced degradation of the PML-RARα fusion protein in NB4 and primary APL cells (Fig 7E).

## Discussion

As_4_S_4_, the major constituent of realgar, is another arsenic drug that has been used in traditional Chinese medicine for many years [16]. Since As^3+^ has been successfully used in APL treatment, the therapeutic potency of As_4_S_4_ in APL treatment has also been investigated [18,19]. The toxicity of ATO to organs, especially the liver and kidney, causes pain to patients [9,10]. As_4_S_4_ has low toxicity and good therapeutic potential for APL treatment, but it could not replace As^3+^. Pastorek M *et al*. have reported that the combination of As_4_S_4_ nanoparticles and As^3+^ induced dose-dependent activation of autophagy and apoptosis in melanoma cell lines [37]. Thus, Low concentrations of As^3+^ (0.1–1.0 μM) in combination with As_4_S_4_ (0.1–1.0 μM) may enhance the therapeutic efficacy of As^3+^ and reduce its adverse effects in the treatment of APL. Herein, we found that As_4_S_4_ and As^3+^ induced the apoptosis and differentiation of NB4 and primary APL cells in dose-dependent manner: 0.5–1.0 μM As^3+^ and 0.5–1.0 μM As_4_S_4_ synergistically promoted cell apoptosis; 0.1–0.25 μM As^3+^ and 0.1–0.25 μM As_4_S_4_ exerted synergistic effects on induction of cell differentiation.

To investigate the mechanism of apoptosis, we examined the effects of combining As_4_S_4_ and As^3+^ on the accumulation of cellular ROS and the expression of Bcl-2, Bax, NFκB, p53, caspase-12 and caspase-3. Mitochondria- and endoplasmic reticulum stress-mediated apoptosis pathways are always triggered by the accumulation of ROS [38,39]. Bcl-2 family members are key in mitochondria-mediated intrinsic apoptosis and caspase-3 activation [38]. The other organelle-controlled apoptosis pathway, endoplasmic reticulum stress-mediated apoptosis, is caused by multiple stimulations [39]. In response to stress, the endoplasmic reticulum induces the activation of caspase-12 and caspase-3, and promotes apoptosis [39]. As_4_S_4_ and As^3+^, the two different forms of arsenic, induced the accumulation of ROS, up-regulated the expression of the pro-apoptotic factor Bax, down-regulated the expression of the anti-apoptotic factor Bcl-2, and induced the activation of caspase-12 and caspase-3. Moreover, the synergistic effect of As_4_S_4_ on As^3+^-induced activation of caspase-12 and caspase-3 was obvious. However, the effects of combining As_4_S_4_ and As^3+^ on cellular ROS accumulation and Bcl-2 and Bax expression were not obvious. In addition to ROS, the p53 tumor suppressor also contributes to mitochondria-mediated and endoplasmic reticulum stress-mediated apoptosis [40,41]. Additionally, the p53 tumor suppressor regulates the G_1_/S transition [24]. As^3+^ and As_4_S_4_ acted synergistically to arrest the cell cycle at G_0_/G_1_ and up-regulate p53 expression. Therefore, both ROS and p53 contributed to the apoptosis of NB4 and primary APL cells induced by As^3+^ and As_4_S_4_. We also studied the effects of combining As^3+^ and As_4_S_4_ on the extrinsic apoptosis pathway [23]. The results of RT-PCR and western-blot suggested that As_4_S_4_ and As^3+^ down-regulated NFκB expression, indicating that As_4_S_4_ and As^3+^ may promote NB4 cell apoptosis by inhibiting NFκB activation [23]. Above all, As_4_S_4_ and As^3+^ showed similar characteristics towards NB4 and primary APL cell apoptosis. Multiple pathways contributed to As_4_S_4_- and As^3+^-induced apoptosis. The combination of As_4_S_4_ and As^3+^ obviously promoted apoptosis by enhancing p53 expression, enhancing the endoplasmic reticulum stress-mediated pathway and inhibiting the NFκB signaling pathway.

Myeloid cell differentiation is regulated by many factors such as the CCAAT/enhancer-binding proteins, PU.1, and *c-Myc* [19]. NB4 and primary APL cells express the PML-RARα oncoprotein that prevents differentiation via the retinoic acid signaling pathway [19,42]. Moreover, As^3+^ was shown to control the fate of the PML-RARα oncoprotein through direct binding to the PML RING domain [8]. Therefore, we analyzed the effects of combining As_4_S_4_ and As^3+^ on the degradation of the PML-RARα oncoprotein in NB4 and primary APL cells. At low concentrations (0.1–0.4 μM), As_4_S_4_ promoted cell differentiation. The combination of 0.2 μM As^3+^ and 0.2 μM As_4_S_4_ obviously enhanced degradation of the PML-RARα oncoprotein and promoted differentiation of NB4 and primary APL cells via the retinoic acid signaling pathway.

## Conclusions

**Fig 8:**
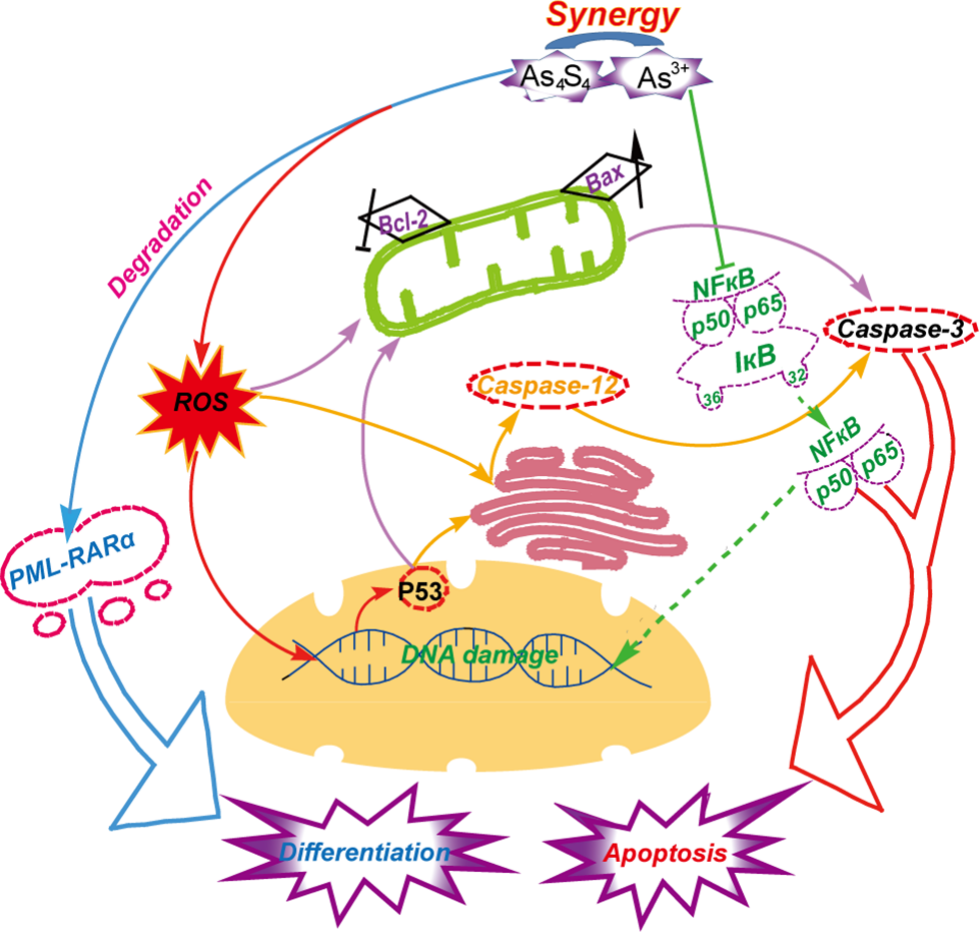
Mechanism for the synergistic effects of As_4_S_4_ and As^3+^ on apoptosis and differentiation of acute promyelocytic leukemia cells.

As_4_S_4_ acts synergistically with As^3+^ towards NB4 and primary APL cell apoptosis and differentiation (Fig 8). As_4_S_4_ and As^3+^ induced the accumulation of cellular ROS and up-regulated the expression of the p53 tumor suppressor. ROS and p53 promoted mitochondria- and endoplasmic reticulum stress-mediated apoptosis by regulating Bcl-2 and Bax expression and inducing activation of caspase-12 and caspase-3. Concomitantly, As_4_S_4_ and As^3+^ synergistically inhibited NFκB activation to promote apoptosis. Moreover, low concentrations of As_4_S_4_ interacted synergistically with As^3+^ to induce degradation of the PML-RARα oncoprotein and promote NB4 and primary APL cell differentiation via the retinoic acid signal pathway. In this work, we found that the combination of As_4_S_4_ and As^3+^ could act synergistically to promote NB4 and primary APL cell apoptosis and differentiation, which may be a better therapeutic avenue for APL treatment.
